# Rheumatologists’ Adherence to EULAR Recommendations for Systemic Sclerosis Treatment: Experience of a Single Center in Serbia

**DOI:** 10.3390/jcm14144994

**Published:** 2025-07-15

**Authors:** Slavica Pavlov-Dolijanovic, Ivan Jeremic, Milan Bogojevic, Zoran Velickovic, Mirjana Zlatkovic-Svenda, Tijana Kojic, Sasa Janjic, Tatjana Dimic, Biljana Stojic, Ana Markovic, Andjela Perunicic, Aleksandra Djokovic, Jelena Petrovic, Nevena Baljosevic, Aleksandar Jankovic, Maja Omcikus, Zorica Terzic Supic, Natasa Milosavljevic, Goran Radunovic

**Affiliations:** 1 Faculty of Medicine, Institute of Rheumatology, University of Belgrade, 11000 Belgrade, Serbia; mirjanazlatkovicsvenda@gmail.com (M.Z.-S.); g.radunovic47@gmail.com (G.R.); 2 Institute of Rheumatology, 11000 Belgrade, Serbia; ivanjeremic@rocketmail.com (I.J.); velickovic.z@yahoo.com (Z.V.); tijanadivljakovic33@gmail.com (T.K.); sasajanjic08@gmail.com (S.J.); tatjana970@yahoo.com (T.D.); b.stojic@yahoo.com (B.S.); anam01944@gmail.com (A.M.); perunicicana@hotmail.com (A.P.); 3 Clinical Center of Montenegro, Department of Rheumatology, 81110 Podgorica, Montenegro; milanbogojevic89@gmail.com; 4 Faculty of Medicine Foca, University of East Sarajevo,73300 Foca, Bosnia and Herzegovina; 5 Faculty of Medicine, University Hospital Medical Center Bezanijska kosa, Department of Cardiology, University of Belgrade, 11000 Belgrade, Serbia; drsaska@yahoo.com; 6 Clinic for Cardiology, University Clinical Center of Serbia, 11000 Belgrade, Serbia; jelenapetrovic2212@gmail.com; 7 Emergency Center, University Clinical Center of Serbia, 11000 Belgrade, Serbia; baljosevic.nevena@yahoo.com; 8 Faculty of Medicine, University Medical Center Zvezdara, Nephrology Department, University of Belgrade, 11000 Belgrade, Serbia; sashajan223@gmail.com; 9 Faculty of Medicine, University Clinical Center of Serbia, Clinic for Pulmonary Diseases, University of Belgrade, 11000 Belgrade, Serbia; majaomcikus@gmail.com; 10 Faculty of Medicine, Institute of Social Medicine, University of Belgrade, 11000 Belgrade, Serbia; zoricaterzic37@gmail.com; 11 Department of Mathematics and Physics, Faculty of Agriculture, University of Belgrade, 11000 Belgrade, Serbia; natasam@agrif.bg.ac.rs

**Keywords:** systemic sclerosis treatment, recommendations, EULAR/EUSTAR, adherence to guidelines

## Abstract

**Background**: The European League Against Rheumatism (EULAR), in collaboration with the European Scleroderma Trial and Research group (EUSTAR), published the first set of treatment recommendations for systemic sclerosis (SSc) in 2009, with subsequent updates in 2016 and 2023. **Objectives**: This study aimed to evaluate how rheumatologists’ clinical approaches to the treatment of SSc evolved following the 2016 update of the clinical management guidelines. **Methods**: Medication use for SSc was analyzed in a cohort of 378 patients. The patients were stratified based on enrollment before (233 patients) and after (145 patients) the guideline update, and medication usage was compared between the two groups. **Results**: Although all patients presented with Raynaud’s phenomenon (RP), only 35% received calcium channel blockers. Medications such as iloprost, phosphodiesterase type 5 (PDE-5) inhibitors, fluoxetine, and bosentan, recommended for the treatment of RP and digital ulcers, were not approved for SSc by the Republic Health Insurance Fund. Treatment for pulmonary arterial hypertension (PAH) was administered to only 16 patients (4.2%), including 2 who received bosentan, 10 who received PDE-5 inhibitors, and 4 who were treated with riociguat. The use of PDE-5 inhibitors increased following the 2016 update of the guidelines. Cyclophosphamide was consistently prescribed for interstitial lung disease (ILD), with an increased frequency observed after the guideline update. No significant differences were observed in the use of methotrexate for skin involvement, ACE inhibitors for scleroderma renal crisis, or antibiotics for gastrointestinal symptoms. Proton pump inhibitors (PPIs) were prescribed to 87.3% of patients with gastrointestinal involvement, with an increase in use of both PPIs and prokinetic agents following the guideline update. **Conclusions**: Rheumatologists’ adherence to the EULAR/EUSTAR guidelines varied considerably, with 25% to 100% of eligible patients receiving the recommended treatments. Concordance improved in the management of PAH, ILD, and gastrointestinal involvement after the 2016 guideline update.

## 1. Introduction

Systemic sclerosis (SSc) is a rare disease characterized by autoimmunity, vasculopathy, and fibrosis of the skin and internal organs. Particularly in the early course, the heterogeneous manifestations of SSc are a challenge for physicians in predicting the development of future severe internal organ involvement and selecting appropriate therapeutic options [1]. The etiology of SSc remains unknown; however, risk factors can be broadly categorized into two main groups: genetic and environmental risk factors (e.g., pollutants, drugs). These factors interact and contribute to key pathological processes, including vascular alterations and immune system dysfunction. SSc-associated vasculopathy is characterized by endothelial apoptosis, which in turn triggers both innate and adaptive autoimmune responses [2]. Damaged endothelial cells release pathogenic antigens that activate Toll-like receptors (TLRs) on dendritic cells. These activated dendritic cells, functioning as professional antigen-presenting cells, subsequently stimulate both cellular and humoral immune responses [3,4]. Activated B and T cells perpetuate endothelial damage, creating a potential vicious cycle in this autoimmune and inflammatory disease. Additionally, immune cells release pathogenic cytokines that promote fibroblast activation [5]. Distinct fibroblast populations have been identified and may serve as predictors of organ involvement severity [6].

The global pooled prevalence of SSc is estimated at 17.6 cases per 100,000 individuals, with a significantly higher incidence in women. The disease exhibits geographic variability, with the lowest prevalence reported in Asia and the highest in both North and South America; however, comprehensive epidemiological data are not available for all continents [7]. Comparative studies of patients from different countries have also highlighted variations in disease severity based on geographic origin [8]. In Serbia, epidemiological data on SSc remain limited. While four university clinical centers are dedicated to the management of SSc patients, a unified national registry has yet to be established.

Systemic sclerosis remains a life-threatening disease. During the acute phase, mortality is primarily associated with renal crisis [9]. As the disease progresses, mortality risk increases due to chronic complications, particularly involving the lungs and heart, as well as disease course and treatment factors [10]. In addition to the cardinal symptoms, SSc patients frequently experience sleep disturbances, which significantly impair their ability to perform daily activities. Poor sleep quality has been shown to adversely affect both overall quality of life and functional capacity [11]. Cognitive impairment is a notable, though under-researched, component of the symptom burden in SSc. Work disability and increased fatigue levels are strongly linked to perceived cognitive dysfunction in this population [12]. More than two-thirds of SSc patients present with at least one comorbidity. The most prevalent comorbid conditions include arterial hypertension, osteoporosis, and dyslipidemia [13]. Both comorbidities and disease activity independently influence the prognosis of SSc patients.

Current treatment approaches focus on managing organ-specific complications, addressing comorbidities, and improving quality of life. Guidelines or recommendations help provide uniform standards in medical practice. When comparing treatment guidelines for SSc from major international organizations (such as the European League Against Rheumatism (EULAR), American Thoracic Society (ATS), Japanese Dermatological Association (JDA), and British Society for Rheumatology (BSR)) [14,15,16,17], we see both similarities in overarching therapeutic strategies and differences based on regional drug approvals, clinical practices, and healthcare systems. The EULAR has been instrumental in developing standardized guidelines for diagnosis and management, promoting research, and fostering collaboration across European countries. In 2009, the EULAR published its first set of recommendations for the treatment of SSc [18]. These guidelines were subsequently updated in 2016 [19] and again in 2023 [14]. The first update was published in November 2016 [19]. A total of 16 updated recommendations (instead of 14 in 2009) address the treatment of several SSc-related organ complications: Raynaud’s phenomenon (RP), digital ulcers (DUs), pulmonary arterial hypertension (PAH), skin and lung disease, scleroderma renal crisis (SRC), and gastrointestinal (GI) involvement. The 2023 guidelines have been expanded to include the treatment of arthritis, which was not addressed in the 2016 recommendations [14]. Most of the new recommendations relate to cutaneous fibrosis and interstitial lung disease (ILD). Despite the availability of established guidelines, there is limited evidence regarding real-world treatment practices for SSc patients. Few data are available on the extent to which rheumatologists adhere to these recommendations in clinical practice.

The aim of this single-center study was to assess the changes in rheumatologists’ clinical approaches to the treatment of SSc before and after November 2016, when the first update of the treatment guidelines was implemented.

## 2. Materials and Methods

The retrospective cohort study included 378 adult SSc patients treated at the Institute of Rheumatology, Belgrade, Serbia, in either inpatient or outpatient settings between January 2010 and April 2021. The inclusion criteria were as follows: patients aged ≥18 years who fulfilled the 2013 American College of Rheumatology (ACR)/EULAR classification criteria or the 1980 classification criteria for SSc. The exclusion criteria were as follows: patients with juvenile SSc; patients with systemic sclerosis sine scleroderma; and patients diagnosed with overlap syndromes (SSc and autoimmune myositis, rheumatoid arthritis, Sjögren syndrome, systemic lupus erythematosus, primary biliary cirrhosis, or autoimmune hepatitis). The diagnosis of SSc was confirmed by a rheumatologist for all patients. Written informed consent was obtained from each participant. The presence of RP and organ involvement, including the peripheral vascular system, skin, respiratory system, cardiovascular system, gastrointestinal system, and urinary system, was documented based on reports from the treating rheumatologist. We collected the data on the presence of RP, DUs (including active, healed, or ever present), inflammatory arthritis, and a history of scleroderma renal crisis. Gastrointestinal involvement was assessed by patient-reported symptoms of dysphagia, gastroesophageal reflux disease (GERD), bloating, and a history of antibiotic use for diarrhea. For the purposes of this study, suspected PAH was defined as an estimated systolic pulmonary artery pressure (PAP) of ≥45 mmHg, based on Doppler echocardiographic assessments of the tricuspid regurgitation jet. The decision regarding which patients would undergo cardiac catheterization was made by cardiologists. The diagnosis of ILD was made when lung pathology was detected on high-resolution computed tomography (HRCT). In cases where HRCT was not performed, ILD was suspected if either (1) a chest X-ray showed increased interstitial markings not due to congestive heart failure and/or (2) a rheumatologist reported physical findings consistent with ILD (dry cough and auscultation findings). In patients with ILD, treatment should be initiated if the results of pulmonary function tests show a corrected diffusing capacity of the lung for carbon monoxide (DLCOc) of <70%, a total lung capacity (TLC) of <70%, and a forced vital capacity (FVC) of <70%.

The data on the patients’ treatment for SSc were analyzed and categorized into two groups: those treated before and those treated after November 2016, corresponding to the publication of the first update of the treatment guidelines. Patients enrolled before November 2016 had their final visit documented on or before that date, while those enrolled afterward had visits documented through April 2021.

Rheumatologists’ adherence to the guidelines was assessed by analyzing the proportion of patients who received targeted therapies for RP, DUs, and skin involvement. Gastrointestinal manifestations were also included, such as GERD, small bowel bacterial overgrowth, pseudo-obstruction, delayed gastric emptying, and dysphagia. Additionally, treatments for renal crisis, PAH, and ILD were evaluated. Medication use was assessed based on the denominator of patients clinically indicated to receive the respective therapy. For example, only patients with a diagnosis of GERD were considered eligible for proton pump inhibitor (PPI) use. Similarly, if ILD was absent, they were not considered for the treatment of ILD.

All statistical analyses were carried out in the SPSS program version 22.0 (SPSS, IBM USA). Descriptive statistics were used to summarize the baseline characteristics of the patients. Chi-square tests, Yates’ chi-square tests, Fisher’s exact tests, and Mann–Whitney U tests were applied as appropriate. A *p*-value of <0.05 was considered statistically significant.

## 3. Results

The baseline characteristics of 378 SSc patients (233 enrolled before November 2016 and 145 enrolled after November 2016) are summarized in Table 1.

The cohort was predominantly female, comprising 91% of patients. The proportion of male patients increased threefold after November 2016 (15%) compared to the period before (5%), although this difference was not statistically significant (*p* = 0.12). Following November 2016, both the mean age at SSc diagnosis and the mean age at onset of RP increased significantly compared to the earlier period (53.21 ± 9.63 vs. 55.8 ± 12.23, *p* = 0.02, and 41.77 ± 11.42 vs. 50.81 ± 13.59, *p* < 0.001, respectively).

Disease duration, defined as the time since the onset of the first non-RP symptoms, was significantly shorter in patients diagnosed after November 2016 (2.29 ± 1.17 years) compared to those diagnosed before November 2016 (11.01 ± 5.73 years). Furthermore, the erythrocyte sedimentation rate (23.66 ± 19.94 vs. 26.63 ± 18.76, *p* < 0.001), the prevalence of non-specific capillaroscopic patterns (5.2% vs. 11%, *p* = 0.04), and the prevalence of ANA positivity (28% vs. 63%, *p* < 0.001) were significantly higher in patients diagnosed after November 2016.

There were no significant differences between the two time points in terms of SSc subtypes (limited cutaneous SSc [lcSSc] and diffuse cutaneous SSc [dcSSc]) or organ involvement, including the joints, gastrointestinal tract, lungs, heart, kidneys, and peripheral vascular system. In contrast, the prevalence of PAH was higher among the patients diagnosed before November 2016 (10% vs. 5%, *p* = 0.01).

The adherence of Serbian rheumatologists to the EULAR/EUSTAR guidelines for the vascular therapeutic continuum in Raynaud’s phenomenon, digital ulcers, and scleroderma renal crisis is presented in Table 2.

All patients in the cohort had RP; however, only 35% received calcium channel blockers (CCBs) for their management. The use of CCBs declined after November 2016, although this difference did not reach statistical significance (41% before vs. 25% after, *p* = 0.13). In Serbia, iloprost, phosphodiesterase type 5 (PDE-5) inhibitors, and fluoxetine are not approved for RP treatment by the Republic Health Insurance Fund.

DUs were observed in 111 of 378 (29%) SSc patients. Recommended treatments for DUs, such as intravenous iloprost and PDE-5 inhibitors, are also not approved for this indication in Serbia. While bosentan has been approved as an effective treatment option for digital ulcers (DUs), its practical use among patients with SSc is significantly limited. This is primarily due to its high cost and the fact that it is not included on the list of medications covered by the Republic Health Insurance Fund, making it financially inaccessible for many patients.

All patients with SRC (10 of 378; 2.6%) were treated with angiotensin-converting enzyme (ACE) inhibitors. The use of ACE inhibitors in SRC remained consistent before and after November 2016. None of the SSc patients required hemodialysis.

The adherence of Serbian rheumatologists to EULAR/EUSTAR guideline-recommended therapies for systemic sclerosis-related organ complications, including pulmonary arterial hypertension and interstitial lung disease, is presented in Table 3.

PAH was suspected in 30 of 378 patients (8%), with confirmation by right heart catheterization (RHC) in 16 cases, representing 4.2% of the total cohort. These patients were treated with vasodilator therapy: 2 received bosentan (an endothelin receptor antagonist), 10 were treated with PDE-5 inhibitors, and 4 received riociguat. The use of PDE-5 inhibitors increased significantly after November 2016 (2.1% prior vs. 3.4% after; *p* = 0.02), likely reflecting the national approval of this therapy for PAH treatment in Serbia.

ILD was identified in 108 out of 378 (28.5%) SSc patients. All affected individuals were treated with cyclophosphamide (CYC), with its use increasing significantly following the first guidelines update (26.2% before vs. 32.4% after, *p* < 0.001). However, hematopoietic stem cell transplantation, recommended for patients with rapidly progressing SSc at risk of organ failure, remains neither available nor approved in Serbia.

The adherence of Serbian rheumatologists to EULAR/EUSTAR guideline-recommended therapies for systemic sclerosis-related organ complications, including those affecting the skin and gastrointestinal tract, is presented in Table 4.

The use of methotrexate (MTX) for skin involvement in early dcSSc remained consistent before and after November 2016 (52% vs. 46%). Nearly all patients with dcSSc (189 of 197; 96%) received MTX, with 122 of 125 (97.6%) treated before November 2016 and 67 of 72 (93%) treated after. This is in line with the EULAR/EUSTAR recommendations, which suggest considering MTX for early diffuse SSc skin manifestations.

The pharmacological management of SSc-related gastrointestinal disorders adhered to the established guidelines. A significant majority of SSc patients (87.3%) were prescribed proton pump inhibitors (PPIs), primarily for treating SSc-related GERD (155 of 378; 41%) and for preventing esophageal ulcers and strictures (175 of 378; 46%). Following the guidelines update in November 2016, PPI usage increased notably. Prokinetic medications were prescribed to only 5.3% of all SSc patients, with prescription rates rising after November 2016 (*p* < 0.001). Intermittent or rotational antibiotic therapy was utilized in 5.3% of SSc patients, with no significant change observed before and after the guidelines update.

## 4. Discussion

The EULAR/EUSTAR has published evidence-based recommendations for the treatment of various manifestations of SSc. Following the initial publication of these recommendations in 2009, only 25% to 40% of SSc patients received the recommended treatment [20]. Pope et al. [21] highlighted a significant gap between guideline compliance and real-world clinical practice, observing that only approximately half of the recommended interventions are consistently implemented. They further noted that, despite widespread expert consensus supporting many of these recommendations, such agreement does not reliably result in adherence to the corresponding treatment protocols in everyday patient care. Vries-Bouwstra et al. [22] identified multiple factors contributing to non-adherence to clinical guidelines, including limited awareness, insufficient familiarity, disagreement with the recommendations, low expectations regarding outcomes, and resistance to changing established clinical practices. In addition, adherence may be influenced by a clinician’s years of experience, especially within a narrowly focused specialty, and by systemic factors such as limited medical resources, restricted access to medications, local drug availability, and national insurance policies. Given these findings, it was of particular interest to assess how SSc is managed at the Institute of Rheumatology in Serbia, especially before and after the 2016 update of the EULAR/EUSTAR recommendations. Accordingly, we evaluated rheumatologists’ adherence to these guidelines in clinical practice within a real-world setting.

Raynaud’s Phenomenon: The 2009 EULAR/EUSTAR guidelines recommend dihydropyridine-type CCBs, particularly oral nifedipine, as the first-line therapy for RP. In 2012, Harding et al. reported that CCBs were underused in the management of systemic sclerosis-associated Raynaud’s phenomenon (SSc-RP), despite existing recommendations. Their analysis of treatment practices across six centers revealed that CCB use ranged from 38.5% to 54.9% [23]. Similarly, in 2016, Moinzadeh et al. [24] reported that in a German cohort of 3248 SSc patients, only 58.1% of those with RP received vasoactive therapy, and of those, just 56.2% were treated with CCBs. These studies concluded that although CCBs were considered the first-line therapy in most centers, they were frequently prescribed at subtherapeutic doses. Nevertheless, the updated 2016 guidelines continued to recommend CCBs as the first-line therapy. At our center, the use of CCBs for the treatment of SSc-RP has been limited in real-world clinical practice, with only 35% of patients receiving them. Notably, a further decline in prescribing rates was observed after November 2016. The reasons for persistently low adherence to SSc-RP treatment guidelines among clinicians, particularly rheumatologists, remain unclear and warrant further investigation. Adverse effects such as hypotension, headache, and lower extremity edema may contribute to this trend. Additionally, during the development of the 2023 guidelines, a systematic literature review group found no new evidence supporting the use of CCBs for the treatment of SSc-RP. As a result, the working group unanimously agreed to retain the previous recommendation without any change in strength or wording. In contrast, fluoxetine was removed from the primary treatment algorithm in the 2023 guidelines and is now listed only as a potential adjunct in selected cases, rather than as a primary therapeutic option [14].

Digital Ulcers: Other vasoactive agents recommended for the treatment of SSc-RP and DUs, such as PDE-5 inhibitors, intravenous iloprost, fluoxetine, and bosentan, are either unavailable or not approved for these indications by the Republic Health Insurance Fund in Serbia. As a result, alternative therapies, such as ACE inhibitors (32.8%) and angiotensin II type 1 receptor antagonists (AT1RAs) (6.6%), are utilized at our institute for the management of SSc-RP and DUs. The existing literature suggests that ACE inhibitors may enhance endothelial function and promote vascular remodeling, while AT1RAs may exert antifibrotic effects in addition to their vasodilatory properties [24]. In Serbia, patients with DUs are also treated with hyperbaric oxygen therapy. Additionally, 178 out of 378 patients (47.1%) in our cohort received ancillary antithrombotic and vasoactive therapies, including aspirin (36.2%) and pentoxifylline (10.8%). The decline in the use of pentoxifylline after 2016 likely reflects the limited evidence supporting its efficacy in SSc. According to experts from the Scleroderma Clinical Trials Consortium and the Canadian Scleroderma Research Group, treatment recommendations for RP vary considerably, with 58% endorsing alternative therapies such as aspirin (75%), topical nitrates (54%), statins (29%), fluoxetine (21%), and pentoxifylline (7%) [25]. In the Serbian cohort, 283 out of 378 SSc patients (74.8%) received some form of vasoactive therapy.

The systematic literature review conducted by the task force in 2023 did not identify any new studies on the effects of intravenous iloprost, PDE-5 inhibitors, or bosentan. Consequently, the task force unanimously agreed to retain the previous 2016 recommendations.

Scleroderma Renal Crisis: Vasoactive drugs, particularly ACE inhibitors, are recommended by the EULAR/EUSTAR guidelines for the treatment of SRC. In our cohort, 10 out of 378 patients (2.6%) experienced SRC, and all were treated with ACE inhibitors, indicating 100% adherence to guideline-based management. ACE inhibitors remain the cornerstone of SRC treatment, as they effectively control malignant hypertension and have been shown to improve survival outcomes. The early initiation of ACE inhibitor therapy can significantly reduce the need for dialysis and improve renal function in affected patients [14]. The 2023 task force reached a high level of agreement to retain this recommendation, which is substantially unchanged. It was also noted that a meta-analysis evaluating the prognosis of SRC in systemic sclerosis (SSc) reported significantly poorer outcomes in patients with prior exposure to ACE inhibitors. For this reason, the recommendation for ACE inhibitor use was not extended to prophylactic (preventive) administration. Additionally, the task force discussed the potential effectiveness of angiotensin II receptor blockers (sartans) in improving SRC outcomes but concluded that studies on this topic are difficult to implement [14].

Pulmonary Arterial Hypertension: The guidelines recommend the use of bosentan, PDE-5 inhibitors, riociguat, intravenous epoprostenol, or other prostacyclin analogs for the treatment of systemic sclerosis-associated pulmonary arterial hypertension (SSc-PAH). In the Serbian cohort, the management of SSc-PAH is primarily undertaken by cardiologists, which may account for the small number of documented cases in our study and the lack of detailed information on specific therapies. Furthermore, financial constraints may have limited access to these high-cost treatments. The 2023 update of the EULAR recommendations for the management of SSc-PAH introduced two new recommendations. First, the combination therapy of a PDE-5 inhibitor and an endothelin receptor antagonist (ERA) is now recommended as first-line treatment. This combination has been shown to reduce the risk of clinical failure by 53%. Second, the task force endorsed a negative recommendation against the use of anticoagulants, particularly warfarin, for the treatment of SSc-PAH.

Systemic Sclerosis Skin Fibrosis: The EULAR/EUSTAR guidelines recommend MTX for the treatment of skin manifestations in early dcSSc. In our study, 96% of patients with dcSSc were treated with MTX (10–17.5 mg per week) as a first-, second-, or third-line therapy. However, the doses administered in our cohort were lower than the 25 mg per week now frequently prescribed to dcSSc patients, despite ongoing uncertainty regarding the efficacy of this higher dosage [26]. At our center, mycophenolate mofetil (MMF) is also included in the management of cutaneous involvement. Unlike the EULAR/EUSTAR recommendation, which primarily emphasizes MTX for skin manifestations, 71% of experts from the Scleroderma Clinical Trials Consortium and the Canadian Scleroderma Research Group support MMF as the first-line treatment. This is followed by MTX, intravenous cyclophosphamide (i.v CYC), and hematopoietic stem cell transplantation, depending on the severity of skin involvement as assessed by the modified Rodnan skin thickness score (mRSS) [25]. The updated 2023 recommendations, in addition to the previously advised MTX, now include MMF and/or rituximab for the treatment of skin fibrosis in SSc. Also, tocilizumab may be considered for the treatment of skin fibrosis in patients with early inflammatory dcSSc [14].

Systemic Sclerosis-Associated Interstitial Lung Disease: For the management of systemic sclerosis-associated interstitial lung disease (SSc-ILD), the EULAR/EUSTAR guidelines recommend CYC, particularly for patients with progressive disease. In our cohort, approximately one-third of patients were diagnosed with SSc-ILD, and the use of CYC increased significantly after November 2016. Additionally, our center has adopted MMF as the first-line therapy, with azathioprine (AZA) frequently used in place of intravenous CYC for the maintenance of remission [27]. The literature suggests that the use of CYC or AZA varies across centers, regardless of the specific definition of ILD included [19,23]. The updated 2023 recommendations, in addition to the previously advised CYC, now include MMF, rituximab, tocilizumab, and/or nintedanib alone or in combination with MMF for the treatment of SSc-ILD [14].

Gastrointestinal Involvement: PPIs are strongly recommended by the EULAR guidelines for the management of gastrointestinal involvement in SSc. In our cohort, over 85% of patients received PPIs both before and after the guideline’s update. PPI use increased during the post-update period, with 46% of patients receiving therapy for the prevention of esophageal ulcers and strictures. The data from the literature indicate that while PPIs are generally effective, they may not provide complete symptom relief in all SSc patients [28]. Moreover, long-term PPI use has been associated with potential adverse effects, including small intestinal bacterial overgrowth, chronic kidney disease, pneumonia, hypomagnesemia, and osteoporosis [29,30]. In our cohort, the prescription of prokinetic agents remains low, although a modest increase was observed after November 2016. A possible reason for their underutilization is limited access to these medications, partly due to safety concerns, such as the risk of long QT syndrome associated with cisapride, which is consistent with findings reported in the literature [31]. Furthermore, the evidence supporting the long-term efficacy of prokinetic agents, such as metoclopramide and domperidone, remains limited. Therefore, their use should be carefully assessed on an individual, case-by-case basis for the management of esophageal dysmotility in SSc patients [32]. Although randomized controlled trials in SSc patients are lacking, the 2023 task force reaffirmed its 2016 recommendation for the use of intermittent or rotating antibiotic regimens in the management of symptomatic small intestinal bacterial overgrowth [14]. This approach remains consistent within our cohort and is supported by the existing literature [14,19,33].

Glucocorticoids: Glucocorticoids (GCs) are not recommended for the treatment of SSc in the 2016 EULAR guidelines due to the risk of serious complications associated with their use. Given the absence of higher-level evidence since 2016, the 2023 task force decided to maintain this recommendation. The task force also expressed strong agreement on the importance of regular blood pressure monitoring when the use of GCs is considered necessary and appropriate. In our cohort, the use of GCs increased after November 2016 from 44% to 53%, although this difference was not statistically significant. The frequent prescription of GCs in SSc, despite limited evidence supporting their efficacy and their known potential for harm, has been well documented in the literature [34]. When used as a part of induction therapy for ILD, GCs are prescribed with caution, and clinical practice varies considerably among experts: 11% report using them consistently, 28% sometimes, 24% occasionally, and 37% avoid them altogether. Among those who do prescribe GCs, a typical regimen includes prednisone at a dose of 20 mg/day, although long-term use is generally avoided [34].

Treatment of Musculoskeletal Involvement in Systemic Sclerosis: The treatment of arthritis was not addressed in the 2016 recommendations. The 2023 guidelines have been expanded to include guidance on the management of arthritis [14]. In our cohort, 45 out of 378 SSc patients (12%) had arthritis, including 39 women and 6 men. These patients were treated with the following therapeutic agents: MTX in 32 cases, antimalarials in 7, and prednisone in 31. Few studies have evaluated the treatment of arthritis in SSc. The pharmacological approach typically includes low-dose corticosteroids, methotrexate, and hydroxychloroquine. Non-tumor necrosis factor biologics, particularly rituximab and tocilizumab, may represent promising options for refractory arthritis [35].

Advantages of Research: Our research shows that, in real life, it is not feasible to strictly follow guidelines from more economically developed regions of the world. Guidelines from major rheumatology societies, such as EULAR and ACR, are read and followed worldwide. However, there is currently a lack of guidelines tailored to economically weaker regions. Therefore, we propose including clinically experienced experts from less developed regions in the guideline development process. We also recommend the creation of specific regional and national guidelines for these parts of the world.

Study Limitations: This study has several limitations. First, some medications are either unavailable or not approved in Serbia, which limits adherence to international treatment guidelines. Second, due to the complexity of the disease and its multisystem involvement, it is often difficult to determine the specific indication for each therapy. For instance, patients with overlapping clinical manifestations, such as RP, DUs, and hypertension, frequently receive multiple vasoactive agents, including CCBs, endothelin receptor antagonists, and aspirin. It was also challenging to determine whether certain treatments were intended for skin, lung involvement, or both. This was particularly true for MTX, where its use for joint symptoms versus systemic disease modification was often unclear. Lastly, treatment decisions may have been influenced by factors such as comorbidities, side effects, dosage, treatment duration, and clinical response.

## 5. Conclusions

A significant gap remains between the recommended and real-world practice in the management of SSc. Although 25% to 100% of our SSc patients received treatments in accordance with guideline recommendations (both basic and updated), overall adherence varied widely. Encouragingly, following the update of the EULAR/EUSTAR guidelines in 2016, there has been an increase in physicians’ adherence to prescribing recommended treatments, particularly for PAH, ILD, and gastrointestinal involvement. However, continued efforts are needed to bridge the gap between the guideline recommendations and clinical practice to ensure optimal care for all SSc patients. Further analysis is warranted in light of the most recent 2023 guideline updates.

## Figures and Tables

**Table 1:** Patients’ baseline characteristics.

Characteristics	All Patients	Patients Enrolled Prior to November 2016	Patients Enrolled After November 2016	*p* Value
Total patients, n (%)	378 (100)	233 (61)	145 (39)	-
Female, n (%)	344 (91)	221 (95)	123 (85)	0.12
Male, n (%)	34 (9)	12 (5)	22 (15)	0.12
Age at diagnosis SSc, mean ± SD, years (range)	54.26 ± 10.76 (18–79)	53.21 ± 9.63 (18–75)	55.8 ± 12.23 (25–79)	0.02
Age at onset RP, mean ± SD, years (range)	45.24 ± 13.04 (16–78)	41.77 ± 11.42 (16–69)	50.81 ± 13.59 (16–78)	0.00
Disease duration from the first non-RP manifestation of SSc, mean ± SD, years (range)	7.66 ± 6.22 (0.6–30)	11.01 ± 5.73 (0.6–30)	2.29 ± 1.17 (0.6–4)	0.00
Disease subset, n (%)				
lcSSc	181 (48)	108 (60)	73 (40)	0.17
dcSSc	197 (52)	125 (63)	72 (37)	0.18
Organ involvement, n (%) of patients				
Arthritis	56 (15)	31 (13)	25 (17)	0.28
Peripheral vascular	111 (29)	87 (37)	24 (17)	0.17
Gastrointestinal tract	155 (41)	101 (43)	54 (37)	0.24
Lung	108 (29)	61 (26)	47 (32)	0.27
Heart	80 (21)	54 (23)	26 (18)	0.23
Kidney	10 (3)	6 (3)	4 (3)	0.25
PAH	30 (8)	23 (10)	7 (5)	0.01
Laboratory findings				
ANA, n (%)	157 (41)	65 (28)	92 (63)	0.02
ACA, n (%)	97 (26)	69 (30)	28 (19)	0.17
ATA, n (%)	61 (16)	25 (11)	36 (25)	0.13
ESR, mean ± SD (range)	24.65 ± 19.60 (3–125)	23.66 ± 19.94 (3–125)	26.63 ± 18.76 (3–90)	0.00
Capillaroscopy pattern, n (%)				
Nonspecific	28 (7.4)	12 (5.2)	16 (11)	0.04
Early	155 (41)	94 (40.3)	61 (42.1)	0.33
Active	156 (41.3)	101 (43.3)	55 (37.9)	0.06
Late	36 (9.5)	26 (11.2)	10 (6.9)	0.05

ACA: Anticentromere antibody; ANA: antinuclear antibody; ATA: anti-topoisomerase antibody; dcSS: diffuse cutaneous systemic sclerosis; ESR: erythrocyte sedimentation rate; lcSSc: limited cutaneous systemic sclerosis; PAH: pulmonary arterial hypertension; RP: Raynaud’s phenomenon; SD: standard deviation; SSc: systemic sclerosis.

**Table 2:** Adherence of Serbian rheumatologists to EULAR/EUSTAR guideline-recommended management strategies for Raynaud’s phenomenon, digital ulcers, and scleroderma renal crisis.

EULAR Guideline (n, %) for Patients with These Characteristics	Guideline-Recommended Medication	All Patients (N = 378)	Patients Prior to November 2016 (N = 233)	Patients After November 2016 (N = 145)	*p* Value
		n (%) of Patients Treated with Guideline-Recommended Therapy			
Raynaud’s phenomenon (378, 100)					
1. Dihydropyridine-type calcium antagonists, should be first-line therapy for SSc-RP	Dihydropyridine-type calcium antagonists	133 (35)	96 (41)	37 (25)	0.13
2. PDE-5 inhibitors should also be considered in treatment of SSc-RP	Not approved for RP in Serbia and cannot be administered				
3. Intravenous iloprost should be considered for severe SSc-RP	Not available or approved in Serbia				
4. Fluoxetine might be considered in treatment of SSc-RP attacks	Not approved for this indication in Serbia				
Digital ulcers (111, 29)					
1. Intravenous iloprost should be considered	Not available or approved in Serbia				
2. PDE-5 inhibitors should be considered	Not approved for this indication in Serbia				
3. Bosentan especially in patients with multiple digital ulcers despite use of calcium channel blockers, PDE-5 inhibitors or iloprost therapy	Not approved for this indication by social insurance fund in Serbia				
Scleroderma renal crisis (10, 2.6)					
1. ACE inhibitors recommended in treatment of scleroderma renal crisis	ACE inhibitors	10 (2.6)	6 (2.6)	4 (2.7)	0.25

ACE: Angiotensin-converting enzyme; NA: not applicable; PDE-5: phosphodiesterase type 5; RP: Raynaud’s phenomenon; SSc: systemic sclerosis.

**Table 3:** Adherence of Serbian rheumatologists to EULAR/EUSTAR guideline-recommended management strategies for pulmonary arterial hypertension and interstitial lung disease.

EULAR Guideline (n, %) for Patients with These Characteristics	Guideline-Recommended Medication	All Patients (N = 378)	Patients Prior to November 2016 (N = 233)	Patients After November 2016 (N = 145)	*p* Value
		n (%) of Patients Treated with Guideline-Recommended Therapy			
Pulmonary arterial hypertension (30, 8)					
1. ERA (Ambrisentan, bosentan, Macitentan) should be considered to treat SSc-related PAH	ERA	2 (0.5)	0 (0)	2 (1.4)	NA
2. PDE5 inhibitors (sildenafil, tadalafil) should be considered to treat SSc-related PAH.	PDE5-inhibitors	10 (2.6)	5 (2.1)	5 (3.4)	0.02
3. Riociguat should be considered to treat SSc-related PAH.	Riociguat	4 (1.1)	0 (0)	4 (2.7)	NA
4. Intravenous epoprostenol should be considered for treatment of patients with severe SSc-PAH (class III and IV).	Not available in Serbia				
5. Prostacyclin analogs (Iloprost, treprostinil) should be considered to treat SSc-related PAH.	Not available in Serbia				
Lung disease (108, 28.5)					
1. Cyclophosphamide should be considered for treatment of SSc-ILD (in particular, SSc with progressive ILD).	CYC	108 (28.5)	61 (26.2)	47 (32.4)	0.00
2. HSCT should be considered for treatment of patients with rapidly progressive SSc at risk of organ failure.	Not approved in Serbia				

CYC: Cyclophosphamide; ERA: endothelin receptor antagonist; HSCT: hematopoietic stem cell transplantation; ILD: interstitial lung disease; NA: not applicable; PAH: pulmonary arterial hypertension; PDE-5: phosphodiesterase type 5; SSc: systemic sclerosis.

**Table 4:** Adherence of Serbian rheumatologists to EULAR/EUSTAR guideline-recommended management strategies for skin and gastrointestinal involvement in systemic sclerosis.

EULAR Guideline (n, %) for Patients with These Characteristics	Guideline-Recommended Medication	All Patients (N = 378)	Patients Prior to November 2016 (N = 233)	Patients After November 2016 (N = 145)	*p* Value
		n (%) of Patients Treated with Guideline-Recommended Therapy			
Skin (378, 100)					
1. Methotrexate may be considered for treatment of skin manifestations of early diffuse SSc.	Methotrexate	189 (50)	122 (52)	67 (46)	NS
SSc-related gastrointestinal disease (155, 41)					
1. PPI should be used for treatment of SSc-related GERD and prevention of esophageal ulcers and strictures.	PPI	330 (87.3)	200 (85.8)	130 (89.6)	0.02
2. Prokinetic drugs should be used for SSc-related symptomatic motility disturbances.	Prokinetic drugs	20 (5.3)	10 (4.3)	10 (6.9)	0.00
3. Intermittent/rotating antibiotics recommended to treat symptomatic small intestine bacterial overgrowth in patients with SSc.		20 (5.3)	10 (4.3)	10 (6.9)	0.17

GERD: Gastroesophageal reflux disease; NS: not significant; PPI: proton pump inhibitor; SSc: systemic sclerosis.

## Data Availability

The datasets generated and/or analyzed during the current study are available from the corresponding author upon reasonable request.
